# Cyclin H expression is increased in GIST with *very-high *risk of malignancy

**DOI:** 10.1186/1471-2407-10-350

**Published:** 2010-07-02

**Authors:** Julian Dorn, Hanno Spatz, Michael Schmieder, Thomas FE Barth, Annette Blatz, Doris Henne-Bruns, Uwe Knippschild, Klaus Kramer

**Affiliations:** 1 Clinic of General-, Visceral- and Transplantation-Surgery, University Hospital Ulm, Ulm, Germany; 2 Clinic of General-, Visceral- and Transplantation-Surgery, Central Hospital Augsburg, Augsburg, Germany; 3 Department of Pathology, University Hospital Ulm, Ulm, Germany; 4 Clinic of Internal Medicine, Klinik am Eichert, Göppingen, Germany

## Abstract

**Background:**

Risk estimation of gastrointestinal stromal tumours (GIST) is based on tumour size and mitotic rate according to the National Institutes of Health consensus classification. The indication for adjuvant treatment of patients with high risk GIST after R_0 _resection with small molecule inhibitors is still a controversial issue, since these patients represent a highly heterogeneous population. Therefore, additional prognostic indicators are needed. Here, we evaluated the prognostic value of cyclin H expression in GIST.

**Methods:**

In order to identify prognostic factors of GIST we evaluated a single centre cohort of ninety-five GIST patients. First, GISTs were classified with regard to tumour size, mitotic rate and localisation according to the NIH consensus and to three additional suggested risk classifications. Second, Cyclin H expression was analysed.

**Results:**

Of ninety-five patients with GIST (53 female/42 male; median age: 66.78a; range 17-94a) risk classification revealed: 42% high risk, 20% intermediate risk, 23% low risk and 15% very low risk GIST. In patients with high risk GIST, the expression of cyclin H was highly predictive for reduced disease-specific survival (p = 0.038). A combination of cyclin H expression level and high risk classification yielded the strongest prognostic indicator for disease-specific and disease-free survival (p ≤ 0.001). Moreover, in patients with tumour recurrence and/or metastases, cyclin H positivity was significantly associated with reduced disease-specific survival (p = 0.016) regardless of risk-classification.

**Conclusion:**

Our data suggest that, in addition to high risk classification, cyclin H expression might be an indicator for "very-high risk" GIST.

## Background

Gastrointestinal stromal tumours (GIST) display a wide range of clinical and pathological features and represent the largest group of mesenchymal tumours of the gastrointestinal tract. They are mostly characterised by a gain-of function mutation of the c-kit gene encoding a receptor tyrosine kinase [1-3]. The original NIH (National Institutes of Health) classification of GIST into four subgroups (very low, low, intermediate or high risk) is based on tumour size and mitotic rate [1] and supplemented by the addition of further parameters [4-7]; (Additional file 1: Table S1). Tumour stage, as well as tumour size, and mitotic rate are relevant markers for clinical outcome of GIST. The combination of large tumour size and/or high mitotic rate is used to identify high risk GIST which is associated with an unfavourable prognosis [1,2]. The identification of genes and gene products correlating with prognosis might have therapeutic implications for further differentiation of high risk GIST in the adjuvant setting. While surgery remains the only curative treatment for GIST, small molecule inhibitors like Imatinib targeting KIT and PDGFR are used as the standard first-line treatment in advanced disease [3,8]. At present, the use of Imatinib in adjuvant treatment is controversial. The suggestion by De Matteo and co-workers generally to use Imanitib in adjuvant therapy of high risk GIST [9,10], has led to pressing demands to introduce additional parameters that help to stratify patients within the high risk GIST group for additional adjuvant therapy concepts [9,10].

Genes involved in cell-cycle regulation, such as cyclins and cyclin-dependent kinases (CDKs), are among such potential markers [11,12]. The impact of deregulation in regard to members of the cyclin-CDK-system on tumourigenesis/tumour progression of GIST has only been evaluated in a few studies [13-15]. There is evidence that a low expression of p27KIP1 (CDKN1B, a cyclin-dependent-kinase-inhibitor) is associated with reduced progression-free survival [16-18]. Furthermore, expression of cyclin A, cyclin B, cyclin D1 and cyclin E seems to be associated with high risk grading but not with clinical outcome [17,19,20]. At present there are no data available regarding the role of cyclin H expression in the progression of GIST. Cyclin H plays a key role in cell cycle regulation by modulating the activity of CDK7 which phosphorylates CDK1, 2, 4 and 6 [21].

In this study, we investigated the expression pattern of cyclin H in a single-centre population of 95 GIST and evaluated its prognostic value, since our gene expression analysis in normal and tumour tissue of a high-risk GIST patient revealed a 10 fold upregulation of cyclin H in tumour tissue.

## Methods

### Human tissue

Medical records as well as paraffin-embedded and frozen specimens of 95 gastrointestinal stromal tumours were included in the study. The clinocopathological features are outlined in Table 1. Informed consent was obtained from all patients. The study was performed with the permission of the independent local ethics committee of the University of Ulm (No. 90/2006 MuZGi).

**Table 1:** Clinicopathologic Features

Initial symptoms (multiple mentions possible)		n	% of 90
No clinical symptoms		25	28%
Pain		33	37%
GI bleeding		25	28%
Anemia		6	7%

**Localization of primary tumour**		**n**	% of **95**
Stomach		58	61%
Small bowel		29	31%
Jejunum		9	9%
Ileum		17	18%
Duodenum		3	3%
Colon		1	1%
Esophagus		2	2%
Others (EGIST, etc.)		5	5%

**Second neoplasias**		**n**	% of **95**
Total		30	32%

		**n**	% of **30**

Colorectal cancer		5	17%
Prostate cancer		4	13%
Breast cancer		3	10%
Gastric cancer		3	10%
Myometrial or cervical cancer		2	7%
Renal or urothelian cancer		2	7%
Pancreatic cancer		1	3%
Others		10	33%

**Histomorphology**		**n**	% of **92**
Spindle cell GIST		80	87%
Epithelioid/Mixed pattern		12	13%
Mixed pattern		11	12%
Epithelioid		1	1%

**Risk of Malignancy **(Fletcher et al.)	**n **(sto/smbo/ot)	**n**	% of **93**
High risk	21/13/5	39	42%
Intermediate risk	13/5/1	19	20%
Low risk	14/7/0	21	23%
Very low risk	8/4/2	14	15%

**sto **= stomach, **smbo **= small bowel, **ot **= others

### Quantification and detection of Cyclin H mRNA

In a pilot study, total RNA was isolated from frozen normal jejunal tissue and from a clinically very aggressive tumour relapse which occurred 1.91 years after the initial diagnosis of a jejunal high risk GIST in a 53-year old female patient (clinical data: age at primary tumour diagnosis: 51 years, features of the primary tumour: size: 9.5 cm, MR: 47 per 50 HPFs; Fletcher classification: high risk of malignancy, no Imatinib treatment prior to resection) using the RNAeasy Kit (Qiagen, Germany). Total RNA (2 μg) was reversely transcribed into complementary DNA using the RT^2 ^First Strand Kit (SuperArray Bioscience Corp., USA). Gene profiling was done as described by the manufacturer using the RT^2 ^profiler PCR array for the human p53 signalling pathway (84 genes, SuperArray Bioscience Corp., USA). For CCNH (gene encoding cyclin H), housekeeping gene HPRT1 (hypoxanthine Phosphoribosyltransferase 1) was used for normalisation. The reactions were carried out in a 7500 Fast Real-Time PCR System (Applied Biosystems, USA). The results were analysed with the 7500 Fast System SDS Software 1.4 (Applied Biosystems, USA).

### Histological evaluation

Original haematoxylin and eosin-stained tumour sections were used to evaluate cell type features and to determine the mitotic rate in 50 high power fields (HPF, magnification: 40×). GISTs were classified according to Fletcher et al. [1] - (see Table 1). Additional risk estimation was performed for all tumours according to the classifications suggested by Miettinen et al. [7], Hornick et al. [4] and Joensuu [6].

### Immunohistochemical analysis

For immunohistochemical analysis the following antibodies were used: anti cyclin H (ab54903, monoclonal, dilution 1:100, abcam, GB), anti KIT (CD117, C-KIT, polyclonal, dilution 1:200, Dako, Glostrup Denmark), anti smooth muscle actin (dilution 1:400, clone 1A4, Dako), anti CD34 (dilution 1:100, clone QBEND10, Dako), anti desmin (D33, monoclonal, 1:10, Linaris, Germany), anti vimentin (Vim3B4, 1:300, Dako), anti NSE (BBC/NC/VI-H14; polyclonal, 1:500, Dako), anti S100 (polyclonal, 1:1000, Dako), anti Ki-67 (clone Mib-1; dilution 1:200, Dako).

In brief, deparaffinised and re-hydrated tissue sections (3 μm) were pretreated in a microwave in CitraPlus solution (Biogenex, USA; 2 minutes on 450 watt then on 80 watt for 20 minutes). After blocking the endogenous peroxidase activity (peroxidase blocking agent, Dako, Denmark), the sections were incubated with the monoclonal mouse anti cyclin H antibody followed by incubation with anti-mouse immunoglobulins conjugated with peroxidase-labeled dextran polymers (N-Histofine, Nichirei Corporation, Japan). Staining was detected with 3, 3'- diaminobenzidine (liquid DAB +, Dako, Denmark) as chromogen and counterstained with hematoxylin before being cover slipped. Cyclin H positivity was defined as positive staining of ≥10% of the tumour cell nuclei according to the modified classifications of Bondi et al. and Kayaselcuk et al. [22,23]. Assessment was done by estimating the rate of positive cells in 10 consecutive fields of view (magnification: 20×) and calculation of the arithmetic mean value of two independent reviewers (TFEB, KK). Results of routine immunhistochemical diagnostics such as expression of CD34, smooth muscle actin, desmin, vimentin and NSE were included in statistical analysis.

### Statistical analysis

For investigation of the obtained data, an exploratory data analysis was performed using SPSS 16 (SPSS Inc., USA) and Excel 2007 (Microsoft Corporation, USA). All criteria were rated equally important, without adjustment of p-values for multiple testing. Tumour size, mitotic rate and age were considered to be continuous variables, while others, such as positivity for immunohistochemical markers, initial symptoms and sex, are treated as categorical variables.

To analyse hypotheses regarding the independence of variables, a contingency table was created and either a chi-square test (X^2^), or (if one or more cells contain less than 5 respondents) Fisher's exact test was performed. Testing was always done two-sided. For statistical analysis of timeline-dependent parameters such as disease-specific survival, a Kaplan-Meier estimation was created and significance was tested using log-rank test. For the calculation of disease-specific-survival (DSS), non GIST-related death-events were censored. P-values < 0.05 were considered statistically significant (α = 0.05). No correction for multiple testing was done.

## Results

A single-centre population of ninety five patients with a mean age of 64.3 years (median 66.9 ± 13.5) ranging from 17 to 94 years and a male/female ratio of 42/53 (44% men, 56% women) underwent surgical resection for a GIST via laparotomy (except one: only biopsy, see below). Clinical manifestations, pathological findings, treatment options, and clinical outcome were evaluated, and statistical analyses were carried out with regard to a potential predictive value of cyclin H.

Major clinical symptoms, tumour location, histomorphology, immunohistology and risk classification are summarised in Table 1 and 2. Tumour size varied between 0.4 and 30.0 centimetres (mean 7.4 ± 5.6) and the mitotic rate per 50 HPF ranged from 0 to 116 (mean 10.8 ± 21). No major differences were obtained by using alternative risk estimations (risk of progressive disease, risk of tumour progression, risk category, according to Miettinen et al. [7], Hornick et al. [4] and Joensuu [6]. All four classification scales did significantly differentiate between high risk and non-high risk GIST (p < 0.001); Table 3.

**Table 2:** Immunohistochemical Results in GIST

Immunohistochemistry	Positive, n (%)		Negative, n (%)		Total, n
c-kit (CD117)	92	(98%)	2	(2%)	94
cyclin H	23	(24%)	72	(76%)	95
CD34	63	(85%)	11	(15%)	74
smooth muscle actin	11	(17%)	53	(83%)	64
desmin	4	(9%)	43	(91%)	47
vimentin	32	(100%)	0	(0%)	32
NSE	6	(55%)	5	(45%)	11
S100	0	(0%)	55	(100%)	55

Two c-kit negative GIST turned to be PDGFRα positive, confirmed as GIST by mutational analysis

**Table 3:** P values regarding different parameters in GIST

Independent Variables	TRD	DSS	DFS	Met/Rec	Count
Tests:	χ2/Fisher Exact	Log-Rank	Log-Rank	χ2/Fisher Exact	(n)
**Sex**	0.180	0.226	0.647	0.626	95

**Localization **(stomach vs. small bowel)	0.671	0.618	0.433	0.611	87
**Tumour size **(≥ 5 cm vs. <5 cm)	**0.005**	**0.008**	**<0.001**	**<0.001**	92
**Tumour size **(≥ 10 cm vs. <10 cm)	**<0.001**	**<0.001**	**<0.001**	**<0.001**	92
**Mitotic rate **(≥ 5/50 HPF vs. <5/50 HPF)	**0.001**	**0.001**	**<0.001**	**<0.001**	90
**Mitotic rate **(≥ 10/50 HPF vs. <10/50 HPF)	**<0.001**	**<0.001**	**<0.001**	**<0.001**	90

**Histomorphology **(spindle vs. not)	0.103	0.099	0.217	0.187	92

**Fletcher **(high vs. non-high)	**<0.001**	**<0.001**	**<0.001**	**<0.001**	93
**Joensuu **(high vs. non-high)	**<0.001**	**<0.001**	**<0.001**	**<0.001**	89
**Miettinen **(high vs. non-high)	**0.001**	**<0.001**	**<0.001**	**<0.001**	86
**Hornick **(high vs. non-high)	**<0.001**	**<0.001**	**<0.001**	**<0.001**	90
**Primary tumour state **(unifocal vs. not)	**<0.001**	**<0.001**	**<0.001**	--	93

**CD34 **(pos vs. neg)	0.680	0.511	0.744	1.000	74
**Aktin **(pos vs. neg)	1.000	0.818	0.844	1.000	64
**Desmin **(pos vs. neg)	1.000	0.930	0.602	1.000	47
**Cyclin H **(pos vs. neg)	0.369	0.189	0.692	0.806	95

**Fletcher **(high vs. non-high)	**<0.001**	**<0.001**	**<0.001**	**<0.001**	93
**Cyclin H **(pos vs. neg, only high-risk)	0.435	**0.038**	0.552	1.000	39
**Cyclin H **(pos high risk vs rest)	**0.004**	**<0.001**	**0.001**	**0.011**	95

**Joensuu **(high vs. non-high)	**<0.001**	**<0.001**	**<0.001**	**<0.001**	89
**Cyclin H **(pos vs. neg, only high-risk)	0.281	**0.009**	0.330	0.730	40
**Cyclin H **(pos. high-risk vs. rest)	**0.004**	**<0.001**	**<0.001**	**0.007**	89

**Miettinen **(high vs. non-high)	**0.001**	**<0.001**	**<0.001**	**<0.001**	86
**Cyclin H **(pos vs. neg, only high-risk)	0.681	**0.050**	0.967	0.696	32
**Cyclin H **(pos. high-risk vs rest)	**0.035**	**<0.001**	**0.012**	0.053	86

**Hornick **(high vs. non-high)	**<0.001**	**<0.001**	**<0.001**	**<0.001**	90
**Cyclin H **(pos vs. neg, only high-risk)	0.306	**0.020**	0.502	1.000	36
**Cyclin H **(pos. high-risk vs. rest)	**0.003**	**<0.001**	**0.001**	**0.007**	90

Dependent on the primary tumour site and the extent of tumour growth, final resection state was R_0 _in 83 (88%), R_2 _in 7 (8%) and R_1 _in 4 (4%) of 94 patients; one 94-year-old patient with a huge gastric GIST was not resected and only biopsies were performed due to a coexisting metastatic obstructive colonic cancer. 16 out of 27 patients who had tumour recurrence and/or metastases were treated with imatinib (200-800 mg/d) in addition to surgery. Of these 15 patients, 8 (50%) achieved stable disease, 4 (25%) attained a partial remission and 4 (25%) had tumour progression. Imatinib did not lead to a complete remission in any of our patients.

### Outcome

**Figure 1:**
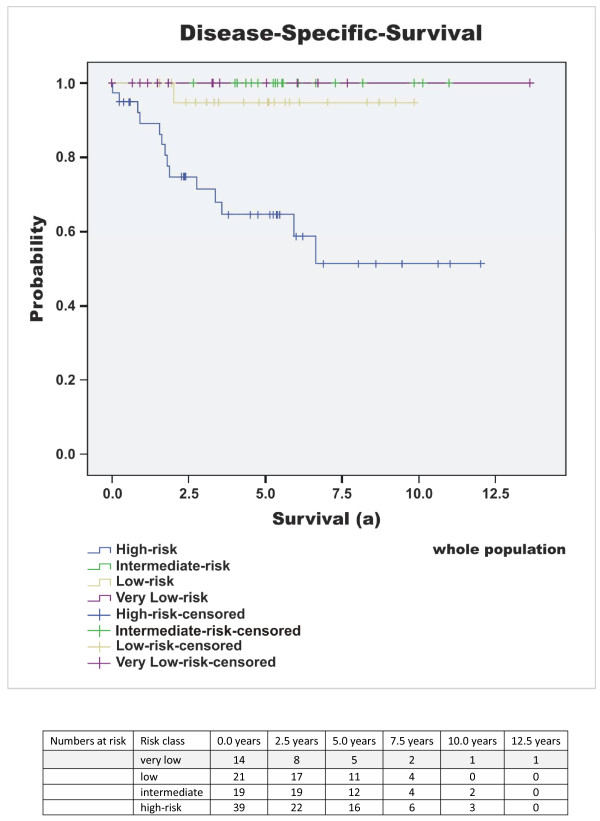
**Disease specific survival - high versus non-high**. Disease specific survival (DSS) of the different risk of malignancy groups according to Fletcher; p < 0.001 (high versus non-high).

**Figure 2:**
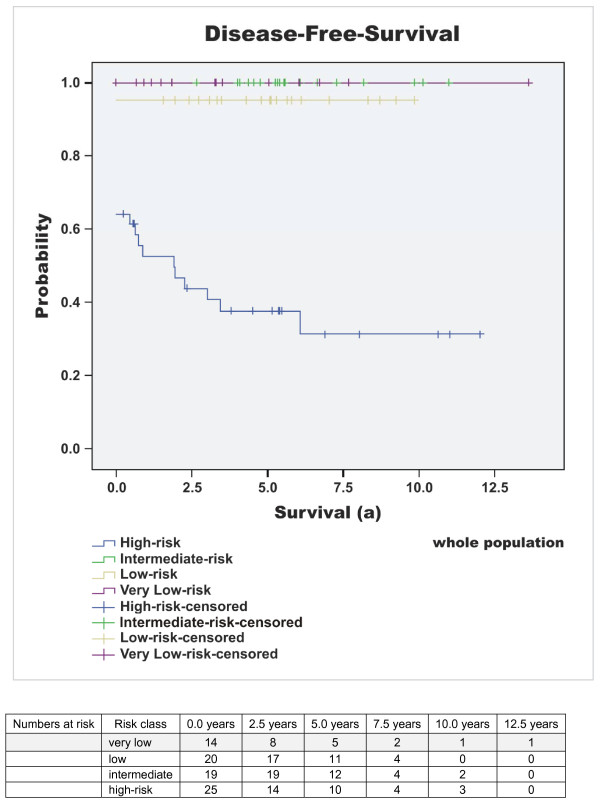
**Disease free survival - high versus non-high**. Disease free survival (DFS) of the different risk of malignancy groups according to Fletcher; p < 0.001 (high versus non-high).

**Figure 3:**
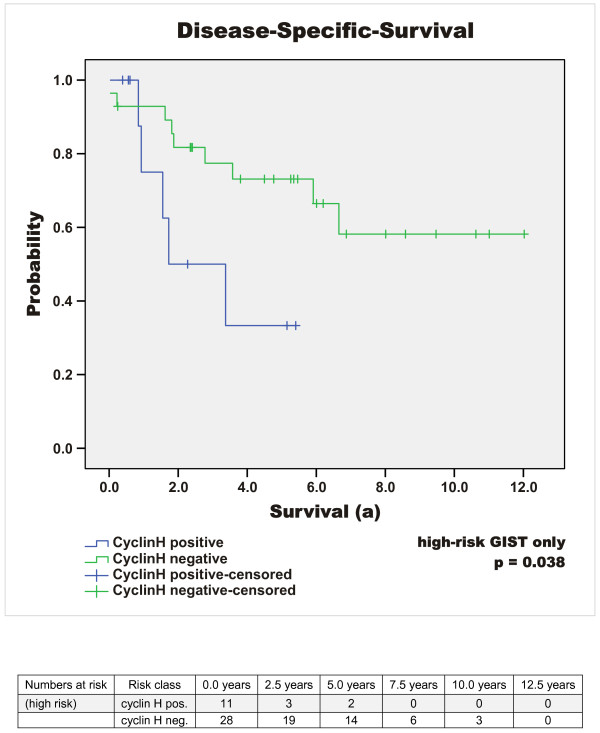
**DSS according to Cyclin H expression in high risk GIST**. DSS in comparison of Cyclin H staining in high risk GIST; positive versus negative (p = 0.038).

**Figure 4:**
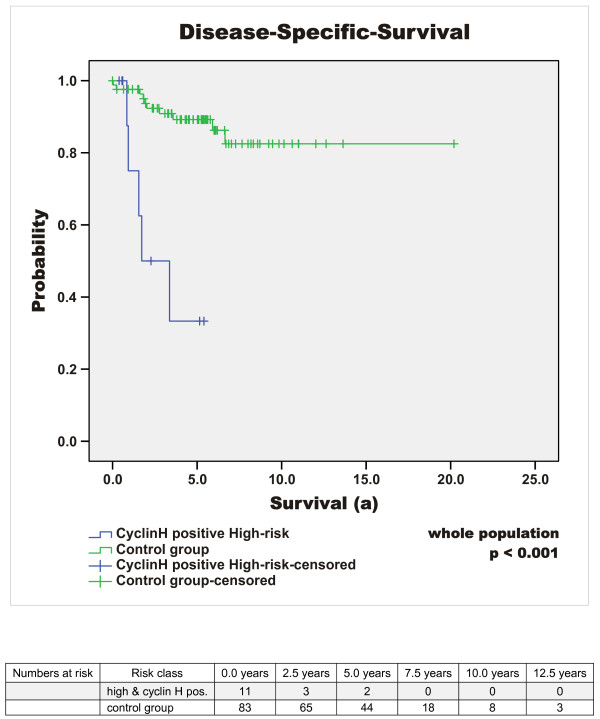
**Cyclin H expression and high risk classification as predictor (DSS)**. Comparison of DSS of all cyclin H positive high risk GIST versus all cyclin H negative high risk GIST as well as all other GIST classified as intermediate, low and very low summarised as control group (p < 0.001).

**Figure 5:**
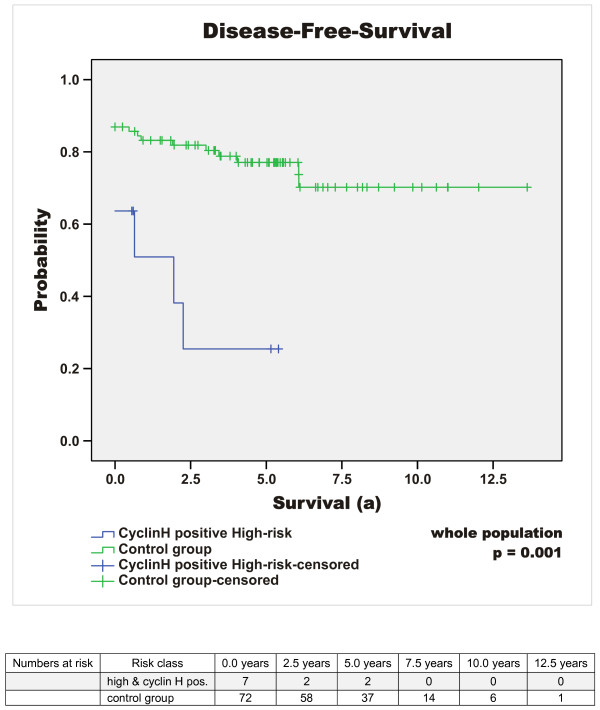
**Cyclin H expression and high risk classification as predictor (DFS)**. Comparison of DFS of all cyclin H positive high risk GIST versus all cyclin H negative high risk GIST as well as all other GIST classified as intermediate, low and very low summarised as control group (p < 0.001).

Median follow-up time of the surviving patients was 5.37 years (64.39 months) (mean 6 years, range 0.4 to 20.2 years). At the time of diagnosis, 15% (n = 14) of all GIST patients showed metastatic disease. 16% (n = 15) of the patients died due to GIST-related causes (overall survival 69% (n = 66)) and the rate of patients with metastases or tumour recurrence increased to 27% (n = 26) during the time of observation. Disease-specific 1-, 3-, and 5-year survival probability (DSS) was 96%, 87%, and 84%. Respectively, disease-free survival (DFS) was 80%, 76% and 72%. The median disease-free interval after primary diagnosis of patients with primary unifocal disease and later developed metastases or tumour recurrence was 2.1 years (25 months), mean 2.6 years (31.5 months), range 0.5 to 6.1 years (5.6 to 73 months). Interestingly, 32% (n = 30) of the patients exhibited additional malignant neoplasms. Concurrent benign neoplasias were found in 17% (n = 16). Results of the survival analysis are summarised in Tables 3, 4, 5 and in Figure 1, 2, 3, 4, 5.

**Table 4:** Results of the Survival Analysis in GIST

*Non-Disease-specific*	1-year	3-year	5-year			
***Overall survival***	**91%**	**79%**	**71%**			

	**1-year DSS (%)**	**3-year DSS (%)**	**5-year DSS (%)**	**1-year DFS (%)**	**3-year DFS (%)**	**5-year DFS (%)**

**Whole cohort**						
*Survival*	96	87	84	80	76	72
**Risk of malignancy **according to Fletcher et al. 2002 [1]						

*Very low*	100	100	100	100	100	100
*Low*	100	95	95	95	95	95
*Intermediate*	100	100	100	100	100	100
*High*	89	71	65	53	44	38
*Non-high*	100	98	98	98	98	98

**Cyclin H**						
*Positive*	90	80	72	78	67	67
*Negative*	97	89	87	80	79	73

**Other criteria**						

*Prim-local*	100	97	94	95	90	85
*Prim-metastasis*	72	33	33	--	--	--
*Met: any time*	84	56	46	27	19	8
*R*_*0 *_*resection*	97	94	90	88	86	85
*R*_*1/2 *_*resection*	83	40	40	33	25	25

DSS = disease specific survival; DSF = disease free survival

### **Cyclin H expression and clinical outcome **(see also Table 3, 4, 5)

**Figure 6:**
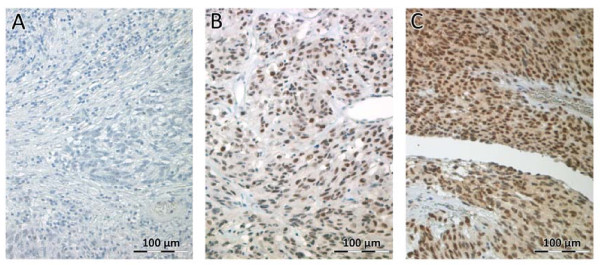
**Expression of cyclin H in gastrointestinal stromal tumours**. Serial sections of gastrointestinal stromal tumours (GISTs) were immunostained using the anti cyclin H specific monoclonal antibody ab54903. **A**: negative staining; **B**: GIST showing a fraction of positively stained cell nuclei; **C**: positivity of almost all cells with strong nuclear and faint cytoplasmic staining.

Quantification of cyclin H expression by Real time PCR in normal intestinal tissue and in a relapse of a jejunal high risk GIST indicated that cyclin H transcription is increased by 10 fold in the tumour tissue. Based on this high transcription level of cyclin H in one GIST, we analysed cyclin H expression at a protein level in the tumour tissue of the same patient. A high nuclear staining of cyclin H was detected. Consequently, immunohistochemical analysis of cyclin H expression in the tumour tissue of 95 GIST patients was undertaken and revealed nuclear positivity of cyclin H in 24% (n = 23) (cut-off value of ≥10% reactive cells) (Figure 6). In the majority of these tumours the intensity was moderate positive (19/23) and only 5 tumours showed a very strong cyclin H staining. Of these five tumours 3 are classified as *low risk*, 1 as *very low risk *and 1 as *high risk *GIST. Further interpretation of strongly positive or moderate positive cannot be assessed because of the limited number of cases. Analysis of the relationship between nuclear cyclin H positivity and risk of malignancy according to Fletcher and co-workers [1] revealed that high risk GIST tumours are 3 times more frequently cyclin H positive than very-low risk GIST (p = 0.176). The disease-specific survival (DSS) of cyclin H positive GIST-patients after 1, 3 and 5 years is approximately 10% below the cyclin H negative cohort (log-rank test, p = 0.189) and within the cyclin H positive GIST patients the disease specific mortality rate is 22% compared to 14% in patients with cyclin H negative GIST (p = 0.369); Table 3. Of the 23 tumours stained positive for Cyclin H the distribution with regard to the different risk categories was as follows: 11 of 39 high risk GIST (28%), 4 of 19 intermediate risk (21%) and 8 of 35 low or very low risk GIST (23%). With focus on the high risk group, cyclin H positivity is significantly related to reduction of disease-specific survival (log-rank: p = 0.038; see Tables 3 &5; Figure 3). In patients who were affected by tumour recurrence or metastases, cyclin H positivity indicated a significantly lower disease-specific survival (e.g. 33.3% vs. 64.2% after 3 years, p = 0.016 log-rank test, see Table 5). The combination of cyclin H positivity and high risk GIST showed the strongest predictive p-value for poor disease-free as well as disease-specific survival (p = 0.001 and p < 0.001, log-rank test; cyclin H positive high risk GIST in comparison with the rest of the whole population; see Table 3 and Figure 5 &6).

**Table 5:** Survival and p-values with focus on subgroups of GIST regarding Cyclin H expression

High-Risk	DSS			DFS		
**Cyclin H**	**positive ** *(n = 11)*	**negative ** *(n = 28)*	all *(n = 39)*	**positive ** *(n = 11)*	**negative ** *(n = 28)*	all *(n = 39)*

**1-year probability (%)**	75	93	89	51	53	52
**3-year probability (%)**	50	77	71	26	49	44
**5-year probability (%)**	33	73	65	26	41	38
**Log-Rank Test (p)**	p = 0.038			p = 0.522		


**Tumour Recurrence or Metastases**	**DSS**			**DFS**		

**Cyclin H**	**positive ** *(n = 7)*	**negative ** *(n = 20)*	all *(n = 27)*	**positive ** *(n = 7)*	**negative ** *(n = 20)*	all *(n = 27)*
**1-year probability (%)**	67	90	84.9	27	30	30
**3-year probability (%)**	33	64	57.0	0	25	19
**5-year probability (%)**	0	58	47.5	0	10	7
**Log-Rank Test (p)**	p = 0.016			p = 0.362		

(results of immunostaining)

No association was found between cyclin H status and localisation of the primary tumour (p = 0.471), sex (p = 0.935), and routine immunohistochemical markers such as CD34 (p = 1.000), smooth muscle actin (p = 0.479), desmin (p = 0.564) and Ki-67 (p = 0.227); Table 3. In multivariate analyses, no statistically relevant related factors could be detected.

## Discussion

The aim of the present study was to evaluate the prognostic value of cyclin H expression in GIST. To this end, the expression of cyclin H was analysed at the mRNA (single case pilot analysis) and consecutively at a protein level of the whole cohort. The cyclin H immunostaining pattern of tumours of 95 patients with GIST was characterised and correlated to clinicopathologic features and clinical outcome.

Due to the absence of reliable genetic or immunohistochemical predictors, tumour size and mitotic rate are still the determining NIH criteria for risk estimation in GIST [1]. Although the classification distinguishes the tumours using a four-point scale (very low, low, intermediate and high risk of malignancy), this scale is only useful to differentiate between high-risk and non-high-risk GIST. High risk classification according to Fletcher et al. [1] is a reliable predictor for the development of metastases or tumour relapse since 85-97% of the affected patients originate from the high risk group. No major differences were found by using alternative risk classification scales according to Miettinen et al., Hornick et al. and Joensuu [4,6,7]. In contrast the high risk group itself remains inhomogeneous based on the period of long-term disease-specific and disease-free survival of GIST patients even without adjuvant treatment (DSS 65%, DFS 38% after 5 years in our study cohort; Table 5; Figure 1 &2). These findings are in line with previous published data and comments highlighting differences up to 100% within this group [9]. In the light of adverse drug effects, the development of secondary resistance to commercial available and broadely used tyrosine kinase inhibitor (TKI) in molecular target based therapy concepts and high annual treatment costs (40,000-80,000 €/year) [3,9,24] it seems mandatory to detect prognostic factors helping to use adjuvant therapy more selectively by identifying patients with a "very-high" risk within the high risk group particularly after an R0 resection.

Quantification of cyclin H mRNA revealed a 10.2 fold increased transcription of cyclin H in a high risk jejunal GIST compared to normal tissue, suggesting an important role of cyclin H and the cyclin-CDK-system in GIST pathogenesis. By immunohistochemical analysis of cyclin H expression in 95 tumour specimens of a single centre population we found a high expression of cyclin H (≥ 10% reactive cells) in 24% of the tumours, which correlated well with the risk of malignancy (p = 0.176). Among all Cyclin H positive patients enrolled in our study there is a tendency for a poor prognosis, although there was no statistical significance (p = 0.692 for DFS and p = 0.189 for DSS). In patients with high risk GIST the expression of cyclin H was highly predictive for the reduction of DSS (p = 0.038). Accordingly, cyclin H expression differentiated high risk and "very-high risk" GIST with regard to disease-specific mortality and might be a valuable tool for further treatment decisions. Moreover, with regard to the whole population, the combination of cyclin H positivity and high risk classification according to Fletcher was strongly predictive of a poor DFS as well as DSS (p = 0.001 and p < 0.001) [1]. Additionally, in patients with tumour recurrence and/or metastases, cyclin H positivity was significantly associated with reduced disease-specific survival (p = 0.016) regardless of risk-classification.

Although the predictive value of Cyclin H is limited with regard to the whole population, our results suggest for the first time a predictive value of cyclin H expression in high risk GIST patients, underlining the importance of the cyclin-CDK system for GIST pathogenesis. Therewith, our data strengthen previous reports on the predictive value of cyclin A, cyclin B, cyclin D1, cyclin E, cdc2, p27 and p21 [16-20,25-27]. However, immunohistochemical positivity of cyclin A, cyclin B, cyclin D1, cyclin E and cdc2 [17,19,20] or the loss of cyclin kinase inhibitors p27 and p21 [16-18,25-27] has only been shown to be associated with high risk grading in GIST, while investigations in regard to clinical outcome are missing. In contrast, our data show a significant correlation of Cyclin H expression with reduced DSS in high risk GIST and in patients with metastases or tumour recurrence.

The efforts to identify parameters that clearly correlate with the clinical outcome of GIST are not restricted to the cyclin-CDK-system. Similarly, the prognostic value of various other factors, such as p53, p16, p21, pRb, E2F1, p27KIP1, Mdm2, Bcl-2 and Bax is not yet entirely clarified. Although changes in their expression have been evaluated in regard to risk ranking, in most cases their correlation with the clinical outcome (DSF, DSS, PFS) has not been validated in detail. Furthermore, the conclusions drawn are still in some cases contradictory [3,17,18,28,29]. Moreover, data relating to the prognostic value of the mutational status are still under debate [30-34]. Altogether, this suggests that one factor does not satisfy the multi-factorial and multidimensional complexity of tumourigenesis and tumour progression of GIST. The identification of factors with a potential prognostic value, as here described for cyclin H, is an important prerequisite for multi-factorial analyses. Simultaneous analysis of cyclin H and a previously investigated factor, p16 [28], within an intersection sub-cohort revealed that the combined positivity of both parameters indicates poor outcome of GIST, irrespective of the mitotic rate or tumour size (p = 0.039) (Additional file 2: Table S2 & S3 and Figure S1). After one year, 50% of the patients with cyclin H- and p16-positive high risk GIST died and none of these patients was tumour free in comparison to 98% survivors and 83% tumour-free patients in the control group. These findings indicate the necessity of multi-factorial follow-up studies (in larger series) for the future.

## Conclusions

In conclusion, conventional risk estimations including tumour size, mitotic rate and tumour location are useful to differentiate high risk and non-high risk GIST. The combination of positivity for cyclin H and high risk classification predicts highly significant poor prognosis in GIST. Also in patients with recurrence or metastases, the expression of cyclin H is the only relevant clinical predictor. Therefore, protein expression of cyclin H may allow subclassification of "very-high risk" (cyclin H-positive high risk GIST) from high risk GIST. Whether cyclin H alone or in combination with any other factor will be an indicator for the necessity of adjuvant treatment of R_0_-resected high risk GIST with a tyrosine kinase inhibitor remains to be further investigated.

## Competing interests

The authors declare that they have no competing interests.

## Authors' contributions

Study concepts: JD, HS, UK, KK; Study design: JD, HS, MS, UK, KK; Data aquisition: JD, HS, MS, TFEB, UK, KK; Quality control of data and algorithms: JD, HS, MS, TFEB, UK, KK; Data analysis and interpretation: JD, HS, MS, AB, TFEB, DHB, UK, KK; Statistical analysis: JD, MS, AB, UK, KK; Manuscript preparation: JD, HS, UK, KK; Manuscript editing: JD, HS, AB, TFEB, DHB, UK, KK; Manuscript review: JD, HS, MS, AB, TFEB, DHB, UK, KK

## Pre-publication history

The pre-publication history for this paper can be accessed here:

http://www.biomedcentral.com/1471-2407/10/350/prepub

## Supplementary Material

Additional file 1 **Table S1**. Table 1: Suggested risk classifications Click here for file

Additional file 2 **Table S2 & S3 and Figure S1**. Table S2: Combined Cyclin H and p16 positivity - Results of the Survival Analysis Table S3: P values for Combined Cyclin H and p16 positivity Figure S1: Disease specific survival in high-risk GIST with combined positivity for cyclin H and p16 (p < 0.001). Click here for file

## References

[B1] Fletcher CD Berman JJ Corless C Gorstein F Lasota J Longley BJ Miettinen M O'Leary TJ Remotti H Rubin BP Diagnosis of gastrointestinal stromal tumors: A consensus approach Hum Pathol 2002 33 459 465 10.1053/hupa.2002.123545 12094370

[B2] Miettinen M Lasota J Gastrointestinal stromal tumors: review on morphology, molecular pathology, prognosis, and differential diagnosis Arch Pathol Lab Med 2006 130 1466 1478 17090188 10.5858/2006-130-1466-GSTROM

[B3] Steigen SE Eide TJ Gastrointestinal stromal tumors (GISTs): a review APMIS 2009 117 73 86 19239429 10.1111/j.1600-0463.2008.00020.x

[B4] Hornick JL Fletcher CD The role of KIT in the management of patients with gastrointestinal stromal tumors Hum Pathol 2007 38 679 687 10.1016/j.humpath.2007.03.001 17437861

[B5] Hou YY Lu SH Zhou Y Xu JF Ji Y Hou J Qi WD Shi Y Tan YS Zhu XZ Predictive values of clinical and pathological parameters for malignancy of gastrointestinal stromal tumors Histol Histopathol 2009 24 737 747 19337972 10.14670/HH-24.737

[B6] Joensuu H Risk stratification of patients diagnosed with gastrointestinal stromal tumor Hum Pathol 2008 39 1411 1419 10.1016/j.humpath.2008.06.025 18774375

[B7] Miettinen M Sobin LH Lasota J Gastrointestinal stromal tumors of the stomach: a clinicopathologic, immunohistochemical, and molecular genetic study of 1765 cases with long-term follow-up Am J Surg Pathol 2005 29 52 68 10.1097/01.pas.0000146010.92933.de 15613856

[B8] Wente MN Buchler MW Weitz J [Gastrointestinal stromal tumors (GIST). Surgical therapy] Chirurg 2008 79 638 643 10.1007/s00104-008-1527-5 18575832

[B9] Hohenberger P Adjuvant imatinib in GIST: a self-fulfilling prophecy, or more? Lancet 2009 373 1058 1060 10.1016/S0140-6736(09)60562-6 19303138

[B10] DeMatteo RP Ballman KV Antonescu CR Maki RG Pisters PW Demetri GD Blackstein ME Blanke CD von Mehren M Brennan MF Adjuvant imatinib mesylate after resection of localised, primary gastrointestinal stromal tumour: a randomised, double-blind, placebo-controlled trial Lancet 2009 373 1097 1104 10.1016/S0140-6736(09)60500-6 19303137 PMC2915459

[B11] Johansson M Persson JL Cancer therapy: targeting cell cycle regulators Anticancer Agents Med Chem 2008 8 723 731 18855574 10.2174/187152008785914833

[B12] Malumbres M Cyclins and related kinases in cancer cells J BUON 2007 12 Suppl 1 S45 52 17935277

[B13] Ali S Role of c-kit/SCF in cause and treatment of gastrointestinal stromal tumors (GIST) Gene 2007 401 38 45 10.1016/j.gene.2007.06.017 17659849

[B14] Bauer S Duensing A Demetri GD Fletcher JA KIT oncogenic signaling mechanisms in imatinib-resistant gastrointestinal stromal tumor: PI3-kinase/AKT is a crucial survival pathway Oncogene 2007 26 7560 7568 10.1038/sj.onc.1210558 17546049

[B15] Tornillo L Terracciano LM An update on molecular genetics of gastrointestinal stromal tumours J Clin Pathol 2006 59 557 563 10.1136/jcp.2005.031112 16731599 PMC1860404

[B16] Shirin H Kravtsov V Shahmurov M Shabat VS Krinshpon I Alin A Avinoach I Avni Y The cyclin-dependent kinase inhibitor, p27, has no correlation with the malignant potential of GIST Digestion 2007 75 4 9 10.1159/000101457 17406118

[B17] Nemoto Y Mikami T Hana K Kikuchi S Kobayashi N Watanabe M Okayasu I Correlation of enhanced cell turnover with prognosis of gastrointestinal stromal tumors of the stomach: relevance of cellularity and p27kip1 Pathol Int 2006 56 724 731 10.1111/j.1440-1827.2006.02038.x 17096729

[B18] Sabah M Cummins R Leader M Kay E Altered expression of cell cycle regulatory proteins in gastrointestinal stromal tumors: markers with potential prognostic implications Hum Pathol 2006 37 648 655 10.1016/j.humpath.2006.01.023 16733203

[B19] Nakamura N Yamamoto H Yao T Oda Y Nishiyama K Imamura M Yamada T Nawata H Tsuneyoshi M Prognostic significance of expressions of cell-cycle regulatory proteins in gastrointestinal stromal tumor and the relevance of the risk grade Hum Pathol 2005 36 828 837 10.1016/j.humpath.2005.03.012 16084954

[B20] Koon N Schneider-Stock R Sarlomo-Rikala M Lasota J Smolkin M Petroni G Zaika A Boltze C Meyer F Andersson L Molecular targets for tumour progression in gastrointestinal stromal tumours Gut 2004 53 235 240 10.1136/gut.2003.021238 14724156 PMC1774925

[B21] Lolli G Johnson LN CAK-Cyclin-dependent Activating Kinase: a key kinase in cell cycle control and a target for drugs? Cell Cycle 2005 4 572 577 15876871

[B22] Bondi J Husdal A Bukholm G Nesland JM Bakka A Bukholm IR Expression and gene amplification of primary (A, B1, D1, D3, and E) and secondary (C and H) cyclins in colon adenocarcinomas and correlation with patient outcome J Clin Pathol 2005 58 509 514 10.1136/jcp.2004.020347 15858123 PMC1770669

[B23] Kayaselcuk F Erkanli S Bolat F Seydaoglu G Kuscu E Demirhan B Expression of cyclin H in normal and cancerous endometrium, its correlation with other cyclins, and association with clinicopathologic parameters Int J Gynecol Cancer 2006 16 402 408 10.1111/j.1525-1438.2006.00407.x 16445666

[B24] Dirnhofer S Leyvraz S Current standards and progress in understanding and treatment of GIST Swiss Med Wkly 2009 139 90 102 19234877 10.4414/smw.2009.12166

[B25] Di Vizio D Demichelis F Simonetti S Pettinato G Terracciano L Tornillo L Freeman MR Insabato L Skp2 expression is associated with high risk and elevated Ki67 expression in gastrointestinal stromal tumours BMC Cancer 2008 8 134 10.1186/1471-2407-8-134 18474118 PMC2396636

[B26] Liu FY Qi JP Xu FL Wu AP Clinicopathological and immunohistochemical analysis of gastrointestinal stromal tumor World J Gastroenterol 2006 12 4161 4165 16830365 10.3748/wjg.v12.i26.4161 PMC4087364

[B27] Yang J Du X Lazar AJ Pollock R Hunt K Chen K Hao X Trent J Zhang W Genetic aberrations of gastrointestinal stromal tumors Cancer 2008 113 1532 1543 10.1002/cncr.23778 18671247 PMC2651090

[B28] Schmieder M Wolf S Danner B Stoehr S Juchems MS Wuerl P Henne-Bruns D Knippschild U Hasel C Kramer K p16 expression differentiates high-risk gastrointestinal stromal tumor and predicts poor outcome Neoplasia 2008 10 1154 1162 18813351 10.1593/neo.08646 PMC2546588

[B29] Schneider-Stock R Boltze C Lasota J Peters B Corless CL Ruemmele P Terracciano L Pross M Insabato L Di Vizio D Loss of p16 protein defines high-risk patients with gastrointestinal stromal tumors: a tissue microarray study Clin Cancer Res 2005 11 638 645 15701851

[B30] Agaimy A Haller F Gunawan B Wunsch PH Fuzesi L Distinct biphasic histomorphological pattern in gastrointestinal stromal tumours (GISTs) with common primary mutations but divergent molecular cytogenetic progression Histopathology 2009 54 295 302 10.1111/j.1365-2559.2008.03214.x 19236505

[B31] Bachet JB Hostein I Le Cesne A Brahimi S Beauchet A Tabone-Eglinger S Subra F Bui B Duffaud F Terrier P Prognosis and predictive value of KIT exon 11 deletion in GISTs Br J Cancer 2009 101 7 11 10.1038/sj.bjc.6605117 19536093 PMC2713701

[B32] Hoeben A Schoffski P Debiec-Rychter M Clinical implications of mutational analysis in gastrointestinal stromal tumours Br J Cancer 2008 98 684 688 10.1038/sj.bjc.6604217 18253129 PMC2259190

[B33] Rubin BP Heinrich MC Corless CL Gastrointestinal stromal tumour Lancet 2007 369 1731 1741 10.1016/S0140-6736(07)60780-6 17512858

[B34] Schildhaus HU Merkelbach-Bruse S Buttner R Wardelmann E Pathology and molecular biology of gastrointestinal stromal tumors (GIST) Radiologe 2009 49 12 1104 8 10.1007/s00117-009-1850-y 19787330

