# Supplementary material for: Cyclin H expression is increased in GIST with very-high risk of malignancy
Source: BMC Cancer. 2010 Jul 2;10:350. doi: 10.1186/1471-2407-10-350 (PMC2916921; doi:10.1186/1471-2407-10-350)
Supplement: Additional file 1 — Table S1. Table 1: Suggested risk classifications [file 1471-2407-10-350-S1.DOC]

Additional file 1

**Table S1: Suggested Risk classifications**

|  |  | **Miettinen et al.** | |  | **Fletcher et al.** |  |  |  |
| --- | --- | --- | --- | --- | --- | --- | --- | --- |
| Mitoses | Size | Gaster | Small Bowel |  |  | Mitoses | Size |  |
| ≤5 |  |  |  |  |  |  |  |  |
|  | ≤2cm | Very Low | Very Low |  | Very Low | <5 | <2cm |  |
|  | >2 and ≤5cm | Low | Low |  | Low | <5 | 2-5cm |  |
|  | >5 and ≤10cm | Low | Intermediate |  | Intermediate | 6-10 | <5cm |  |
|  | >10 | Intermediate | **High** |  |  | <5 | 5-10cm |  |
| >5 |  |  |  |  | **High** | >5 | >5cm |  |
|  | ≤2cm | Low | **High** |  |  | >10 | Any |  |
|  | >2 and ≤5cm | Intermediate | **High** |  |  | Any | >10cm |  |
|  | >5 and ≤10cm | **High** | **High** |  |  |  |  |  |
|  | >10 | **High** | **High** |  |  |  |  |  |
|  |  |  |  |  |  |  |  |  |
|  |  | **Hornick et al.** | |  | **Joensuu** |  |  |  |
| Mitoses | Size | Gaster | Small Bowel |  |  | Mitoses | Size | Site |
| <5 |  |  |  |  | Very Low | ≤5 | <2cm | Any |
|  | ≤2cm | Very Low | Very Low |  | Low | ≤5 | >2 and ≤5cm | Any |
|  | >2 and ≤5cm | Very Low | Low |  | Intermediate | >5 | >2 and ≤5cm | Gastric |
|  | >5 and ≤10cm | Low | Intermediate |  |  | 6-10 | <5cm | Any |
|  | >10 | Intermediate | **High** |  |  | ≤5 | >5 and ≤10cm | Gastric |
| ≥5 |  |  |  |  | **High** | Any | >10cm | Any |
|  | ≤2cm | Very Low | Intermediate |  |  | >10 | Any | Any |
|  | >2 and ≤5cm | Intermediate | **High** |  |  | >5 | >5cm | Any |
|  | >5 and ≤10cm | High | **High** |  |  | >5 | >2 and ≤5cm | Nongastric |
|  | >10 | High | **High** |  |  | ≤5 | >5 and ≤10cm | Nongastric |
|  |  |  |  |  |  | Any | Any | Tumor rupture |
