# Supplementary material for: Cyclin H expression is increased in GIST with very-high risk of malignancy
Source: BMC Cancer. 2010 Jul 2;10:350. doi: 10.1186/1471-2407-10-350 (PMC2916921; doi:10.1186/1471-2407-10-350)
Supplement: Additional file 2 — Table S2 & S3 and Figure S1. Table S2: Combined Cyclin H and p16 positivity - Results of the Survival Analysis Table S3: P values for Combined Cyclin H and p16 positivity Figure S1: Disease specific survival in high-risk GIST with combined positivity for cyclin H and p16 (p < 0.001). [file 1471-2407-10-350-S2.DOC]

Additional file 2

**Table S2. Combined Cyclin H and p16 positivity Results of the Survival Analysis**

|  | **1-year DSS (%)** | **3-year DSS (%)** | **5-year DSS (%)** | **1-year DFS (%)** | **3-year DFS (%)** | **5-year DFS (%)** |
| --- | --- | --- | --- | --- | --- | --- |
| **Whole cohort** |  |  |  |  |  |  |
| *Survival* | 96 | 87 | 84 | 80 | 76 | 72 |
|  |  |  |  |  |  |  |
| **Cyclin H & p16** |  |  |  |  |  |  |
| *Positive* | 83 | 66 | 66 | 64 | 64 | 64 |
| *Negative* | 97 | 90 | 87 | 82 | 78 | 73 |
|  |  |  |  |  |  |  |
| **Cyclin H & p16 in high risk** | | | | | | |
| *Pos. in high risk* | 50 | 0 | 0 | 0 | 0 | 0 |
| *Neg. in high risk* | 94 | 80 | 73 | 60 | 50 | 43 |
|  |  |  |  |  |  |  |
| **High-risk Cyclin H & p16 positive vs rest** | | | | | | |
| CH+P16 pos high | 50 | 0 | 0 | 0 | 0 | 0 |
| *rest* | 98 | 91 | 88 | 83 | 79 | 77 |
|  |  |  |  |  |  |  |
| **Met /Rec Cyclin H & p16** positive vs neagative | | | | | | |
| *positive* | 67 | 33 | 0 | 0 | 0 | 0 |
| *negative* | 90 | 64 | 58 | 36 | 23 | 9 |

P16 staining and analysis was performed in cooperation and according to Schmieder et al. 2008 [27]

Additional file 2

**Table S3: P values for Combined Cyclin H and p16 positivity**

| **Independent Variables** | **TRD** | **DSS** | **DFS** | **Met/Rec** | **Count** |
| --- | --- | --- | --- | --- | --- |
| Tests: | χ2 / Fisher Exact | Log-Rank | Log-Rank | χ2 / Fisher Exact | (n) |
|  |  |  |  |  |  |
| **Cyclin H & p16**  (pos vs. neg, whole population) | 0.231 | **0.039** | 0.361 | 0.550 | 93 |
| **Cyclin H & p16**  (pos vs. neg, only high‑risk **ROM**) | 0.163 | **<0.001** | **0.018** | 0.376 | 39 |
| **Cyclin H & p16**  (cyclin H & p16-pos. high-risk **ROM** vs. rest) | **0.006** | **<0.001** | **<0.001** | **0.006** | 91 |
| **Cyclin H & p16**  (pos vs. neg, only Rez/Met) | 0.342 | **<0.001** | 0.062 | — | 27 |

**TRD**=Tumor-Related Death, **DSS**=Disease specific survival, **DFS**=Disease free survival, **Met/Rec**=Metastases or Recurrence **ROM** = risk of malignancy (Fletch*er et* al, 2002) [1] 16 staining and analysis was performed in cooperation and according to Schmieder et al. 2008 [27]

Additional file 2

**Figure S1**: Disease specific survival in high-risk GIST with

combined positivity for cyclin H and p16 (p<0.001).
